# Immunoinformatics Identification of B- and T-Cell Epitopes in the RNA-Dependent RNA Polymerase of SARS-CoV-2

**DOI:** 10.1155/2021/6627141

**Published:** 2021-04-20

**Authors:** Niti Yashvardhini, Amit Kumar, Deepak Kumar Jha

**Affiliations:** ^1^Department of Microbiology, Patna Women's College, Patna 800 001, Bihar, India; ^2^Department of Botany, Patna University, Patna 800 005, Bihar, India; ^3^Department of Zoology, P. C. Vigyan Mahavidyalaya, Chapra, Bihar 841 301, India

## Abstract

SARS-CoV-2 (Severe acute respiratory syndrome coronavirus-2) is a newly emerged beta coronavirus and etiolating agent of COVID-19. Considering the unprecedented increasing number of COVID-19 cases, the World Health Organization declared a public health emergency internationally on 11^th^ March 2020. However, existing drugs are insufficient in dealing with this contagious virus infection; therefore, a vaccine is exigent to curb this pandemic disease. In the present study, B- and T-cell immune epitopes were identified for RdRp (RNA-dependent RNA polymerase) protein using immunoinformatic techniques, which is proved to be a rapid and efficient method to explore the candidate peptide vaccine. Subsequently, antigenicity and interactions with HLA (human leukocyte antigen) alleles were estimated. Further, physicochemical properties, allergenicity, toxicity, and stability of RdRp protein were evaluated to demonstrate the specificity of the epitope candidates. Interestingly, we identified a total of 36 B-cell and 16 T-cell epitopes using epitopes predictive tools. Among the predicted epitopes, 26 B-cell and 9 T-cell epitopes showed non-allergenic, non-toxic, and highly antigenic properties. Altogether, our study revealed that RdRp of SARS-CoV-2 (an epitope-based peptide fragment) can be a potentially good candidate for the development of a vaccine against SARS-CoV-2.

## 1. Introduction

Severe acute respiratory syndrome coronavirus-2 (SARS-CoV-2) is the causal agent of the current pandemic of novel coronavirus disease 2019 (COVID-19), inducing moderate to severe respiratory illness in humans across the globe [1]. The disease has originated from the wet seafood market in Wuhan city of Hubei province (China), in late December 2019 [2]. COVID-19 has now affected 218 countries, posing a significant public health threat around the world. As of February 13^th^, 2021, worldwide 107,838,255 confirmed cases of COVID-19 have been reported to WHO including 2,373,398 casualties (WHO COVID-19 Dashboard).

SARS-CoV-2 is an enveloped single-stranded positive-sense RNA virus of 30 kb length [3]. The genome of SARS-CoV-2 is comprised of 14 ORF sequences, encoding 29 proteins which include four structural proteins such as S (spike), E (envelope), M (membrane), and N (nucleocapsid) proteins. Additionally, the genome of this virus encodes 16 non-structural proteins (nsp) and 9 accessory proteins [4, 5] including viral main replication/transcription mediating protein, the RNA-dependent RNA polymerase (RdRp) (also called nsp12). RdRp of SARS-CoV-2 is a conserved protein and is considered as a key determinant for the RNA viruses because of its role in viral genome replication/transcription and also the absence of its homolog in host cells makes this protein a suitable candidate for the development of a peptide vaccine against SARS-CoV-2 [6, 7].

Peptides are considered as a crucial vaccine candidate, owing to their easy production, chemical stability, and inability to cause infections. Previous studies have shown that the immunoinformatics strategies have been proved to be effective against several viral diseases, such as SARS-CoV [8], influenza [9], zika virus [10], yellow fever [11], including hepatitis B, and foot and mouth virus diseases [12, 13]. *In vitro* culture of pathogenic microorganisms is not required for the design of epitope-based peptide vaccine; therefore, these vaccines are found to be biologically safe and their selectivity induces accurate immune responses [14, 15]. These vaccine candidates can be designed to encompass multiple determinants from various pathogens or multiple epitopes from the same pathogenic organism. *In silico* epitope-based vaccines have a remarkable advantage over the conventional methods of vaccine development because they are found to be highly specific, capable of avoiding unwanted immune responses, eliciting long-lasting innate and adaptive immunity, safe and less time-consuming, and are reasonably cheaper [16].

In the present study, we have attempted to design a multi-epitope potential peptide vaccine candidate for SARS-CoV-2 using an immunoinformatics approach. Epitopes were predicted for B-cell, T-cell, and MHC I to design a multi-epitope vaccine construct. Further, the physicochemical characteristics, allergenicity, toxicity, hydropathy index, and stability of the RdRp protein were estimated to demonstrate the specificity of the epitope candidates. Thus, in this study, we tried to design an efficacious multi-epitope vaccine utilizing the tools of immunoinformatics to generate both B-cell and T-cell immune responses to curb SARS-CoV-2 infections. However, information regarding the use of nsp like RdRp as a vaccine candidate against SARS-CoV-2 is still scanty. Moreover, to obtain the high efficacy of the designed epitope vaccine, further *in vitro* and *in vivo* studies are necessary to validate immunogenicity and safety, the major concern of a designed candidate vaccine.

## 2. Methods

**Figure 1:**
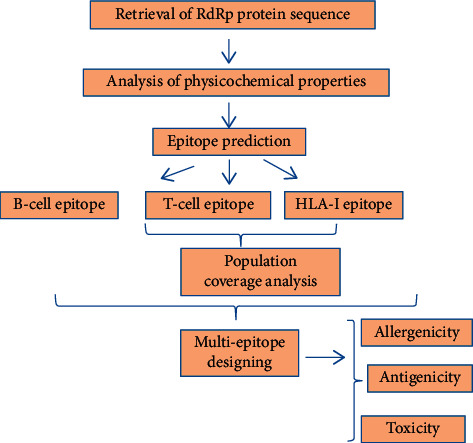
The workflow showing the different approaches used for epitope based peptide vaccine prediction.

The workflow showing the steps for the epitope-based peptide vaccine prediction used by us is shown in Figure 1.

### 2.1. Collection of Protein Sequences

The full-length protein sequence of SARS-CoV-2 RdRp protein was retrieved from the NCBI virus database.

### 2.2. Analysis of Physicochemical Properties of RdRp Protein

The physicochemical properties of the RdRp protein were estimated using the Protparam tool of ExPASy online web server. Protparam is a tool that computes several physical and chemical parameters of a query protein sequence. Protparam estimates the molecular weight, instability index, extinction coefficient, estimated half-life, and the amino acid composition of proteins. The hydropathy plot of the RdRp protein was created using ProtScale tool of ExPASy [17]. The ProtScale computes and represents the profile produced by any amino acid scale on a query protein. These scales include hydrophobicity scales and the secondary structure conformation parameters. Prosol server was used for solubility analysis of RdRp protein *in silico*. Protein-sol performs theoretical calculations and predicts algorithms to calculate protein solubility and stability.

### 2.3. Identification of Linear B-Cell Epitopes and IFN-Inducing Epitopes

The prediction of linear B-cell epitopes was done using Immune Epitope Database (IEDB) [18] as well as Bepipred 2.0 [19]. This web server predicts epitopes based on parameters like flexibility, accessibility, hydrophilicity, turns, polarity, and the antigenic propensity of the protein using amino acid scales and HMMs. For this analysis, the FASTA sequence of RdRp protein was the targeted protein fed as input in the server, and analysis was done with default parameters. Moreover, interferon-inducing epitopes were predicted using the IFNepitope server to detect the peptides capable of inducing interferons with SVM based strategy of prediction [20].

### 2.4. Prediction of SARS-CoV-2 T-Cell Epitopes and MHC Allele Identification

IEDB [18] Tepitool server was used to predict the T-cell epitope binding along with the detection of the MHC allele showing the highest affinity for the T-cell epitope. This web server predicts epitopes restricted to a large number of MHC I and MHC II alleles.

### 2.5. Population Coverage and Conservation across Antigen

To detect the global population coverage of the HLA allele, the Population Coverage tool of the IEDB server was used [18]. Population Coverage Analysis (PCA) predicts the probability of response of each peptide in different countries around the world based on HLA matching. This tool estimates the number of individuals predicted to respond to a provided epitope with known MHC restrictions.

### 2.6. Antigenicity and Allergenicity Evaluation

The antigenicity of the RdRp protein was estimated using the Vaxijen v2.0 server which predicts antigens according to the auto cross-covariance (ACC) transformation of the protein sequences [21]. The prediction of vaccine allergenicity is important, as a good vaccine needs to be non-allergenic. The allergenicity of the RdRp protein was predicted using the AllerTOP server, which evaluates protein allergenicity on autocross variance (ACC method) that explains residues hydrophobicity, size, flexibility, and other parameters [22].

## 3. Results

### 3.1. Collection of Target Protein Sequences

RdRp protein sequence (932 amino acid long) of SARS-CoV-2 Indian isolate was retrieved from the NCBI virus database. This protein sequence was used in this study for predicting epitopes.

### 3.2. Estimation of Physicochemical Properties and Hydropathy Index of SARS-CoV-2 RdRp Protein

**Figure 2:**
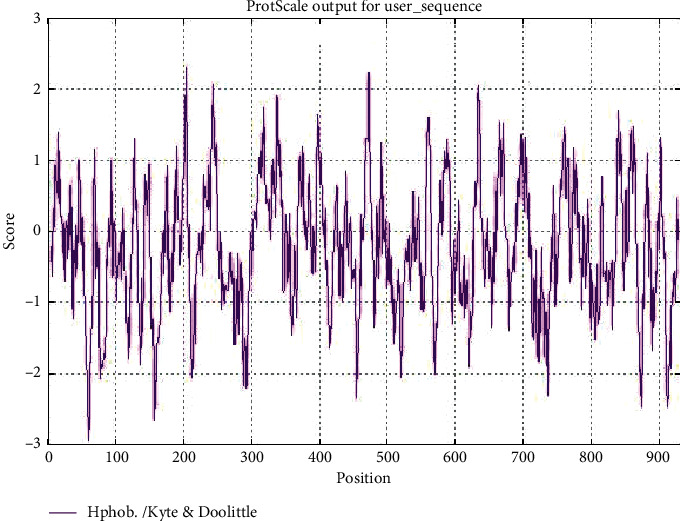
Hydropathy plot of SARS-CoV-2 RdRp protein as estimated by Protscale tool of ExPASy.

The physicochemical properties of RdRp protein were estimated using Protparam (ExPASy) which revealed that the RdRp protein is 932 amino acids in length with a molecular weight of 106660.24 Da, the total number of atoms is 14771, aliphatic index 78.43, instability index 28.32, and GRAVY score of −0.224. It can also form hydrogen bonds and hence is a stable protein (Table 1). The hydropathy plot showed N-terminal amino acid to be more hydrophobic as compared to the C-terminal end (Figure 2).

### 3.3. Identification of B-Cell Epitope

**Figure 3:**
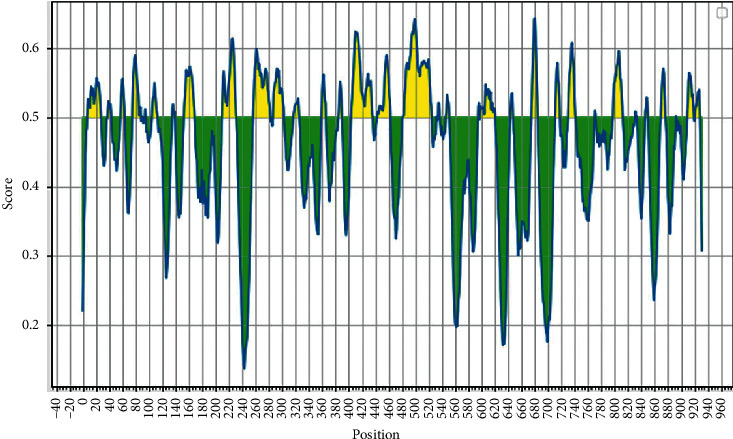
B-cell epitope prediction of RdRp protein sequence. The threshold cutoff is 0.4, above which the residues are epitopes.

B-cell linear epitopes for the SARS-CoV-2 RNA-dependent RNA polymerase protein were predicted using IEDB server with full-length RdRp protein sequence as query and a threshold value of 0.4 was selected. A total of 36 linear B-cell epitopes were predicted for RdRp protein which are placed in Table 2 with their sequence, region, antigenicity, and allergenicity (Table 2). Out of the 36 B-cell epitopes, 10 were allergenic while others were non-allergenic as well as highly immunogenic. In Figure 3, yellow peak represents the epitopic regions whereas the green color slopes denote the non-epitopic region. Figure 3 shows B-cell epitope prediction of RdRp protein sequence. The threshold cutoff is 0.4, above which the residues are epitopes.

### 3.4. T-Cell Epitope Prediction and Identification of MHC Binding Allele

We identified 16 T-cell binding epitopes showing different allele binding affinity. The sequence of these epitopes along with their position is shown in Table 3. Moreover, MHC allele binding affinity was also predicted which showed the binding with MHC class I molecules (Table 4). These epitopes can induce immunogenicity and hence increase cytokine production in cells to reduce the infection.

### 3.5. Population Coverage Analysis

The T-cell epitopes were subjected to Population Coverage Analysis which is requisite for MHC molecules, being polymorphic in nature. This tool enhances the understanding of the fraction of individuals responding towards the epitope predicted for any antigen. We selected 15 geographical areas which were East Asia, Northeast Asia, Southeast Asia, South Asia, West Asia, East Africa, West Africa, South Africa, North Africa, West Indies, Central Africa, North America, South America, Central America, and Europe. This tool predicted average population coverage of 92.5% for MHC class-I binding peptide fragments.

### 3.6. Assessment of Antigenicity and Allergenicity

To predict the antigenicity of the RdRp protein, the VaxiJen v2.0 server was used. The property of antigenicity relies on the ability of the vaccine to bind to both the B-cell and T-cell receptors and increase the immune response in the host. The antigenicity analysis indicated the antigenicity of the epitopes to be 0.620516 with a threshold of 0.4%, above which the epitopes were called antigenic (Tables 2 and 3). The predicted T-cell epitope peptide sequence PDILRVYANLGERVRQALLKTVQFCDAMRNAGIVGVLTLDNQDLNGNWYDFGDFIQTTPG was found to be highly immunogenic (score 0.92) followed by YWDQTYHPNCVNCLDDRCILHCANFNVLFSTVFPPTSFGPLVRKIFVDGVPFVVSTGYHF with a score of 0.65.

A peptide to be used as a vaccine, it must be a non-allergen to the host system. The allergenicity of both the B-cell and T-cell epitopes were predicted in which only seven T-cell epitopes were found to be allergen while other nine were non-allergen and hence can be used as a construct in vaccine production (Tables 2 and 3). A total of 26 B-cell epitopes were found to be non-allergenic and therefore considered as a potent vaccine target. Based on the above findings, the B-cell epitopes with sequences GNKIADK, NLHSSRL, and EKDEDDN are highly immunogenic as well as non-allergenic and can be used as a candidate vaccine target. Overall, RdRp is an antigenic as well as non-allergenic protein (except few epitopes) and therefore can be used as a potential vaccine candidate against SARS-CoV-2 infections.

## 4. Discussion

The rapid emergence of COVID-19 has become a major challenge to public health across the globe. Therefore, antiviral therapeutics are urgently needed to combat SARS-CoV-2 infections. Primarily, the virus causes severe respiratory infection and pneumonia in humans, which is manifested by dry cough, sore throat, fever, and dyspnea (shortness of breath). The symptoms of the SARS-CoV-2 infection begin within 2 days or it may last up to 14 days after exposure; transmission of disease may occur through human-to-human contact or from the infected inanimate matters (based on the recommendation of centre for disease control and prevention) [23–25].

The design and development of epitope-based peptide vaccines using various predictive tools of immunoinformatics have become a research priority nowadays. The conventional method of potent vaccine development requires an extensive investigation, identification, and establishing an immunological correlation with the SARS-CoV-2. Generally, the mechanism behind the synthesis of peptide vaccine involves the identification of immunodominant B-cell and T-cell epitopes that are able to induce specific immune responses. Furthermore, for a peptide vaccine to be immunogenic substantially, its B-cell epitope of a target molecule must be linked with a T-cell epitope. The T-cell epitopes commonly consist of 8–20 amino acids (small peptide fragments) and are found to be more propitious and hence generate long-lasting immune response mediated by CD8^+^ T-cells [26] while the B-cell epitope is made up of a linear chain of amino acid that can be a protein [27, 28]. The experiment-based vaccine development procedure is found to be lengthy and expensive which takes several years with the significantly high rate of failure to develop a commercially effective vaccine. However, the predicted *in silico* epitope-based peptide vaccine might be a potentially good candidate against SARS-CoV-2.

In the present research work, we investigated the RdRp protein, as a potential immunogenic epitope that induces robust and long memory of humoral (B cell) as well as cell-mediated (T-cell) immune response, and serves as a potent vaccine candidate. The efficacy of vaccines mainly depends on the selection of antigen molecules [28]. It is evident from the previous studies that the S (surface) glycoprotein of SARS-CoV-2 has rapidly become a potential target of vaccine design using the immunoinformatics approach [29, 30]. However, the non-structural protein like RdRp is essential for the replication/transcription of this virus. Furthermore, nsps are considered as a more conserved protein as compared to structural proteins of SARS-CoV-2 [31, 32]. Approximately, two-thirds of the SARS-CoV-2 genome is made up of nsps. Johnson et al. (2007) have reported that nsps are found highly expressed at the site of infection and presented by their MHC-I from the beginning (1^st^ day) of infection [33, 34]. It has been observed that nsps show significantly lower glycosylation density as compared to structural proteins. Therefore, B-cell and T-cell epitopes of RdRp protein could be used as an effective and promising vaccine candidate to fight against SARS-CoV-2 infections, as earlier studies showed negative effects of dense glycosylation of epitopes and its subsequent recognition by T-cells [35–37].

Predicted MHC class-I epitopes were found interacting with the various HLA alleles and seem to be antigenic. The physicochemical properties and the hydropathy index of the RdRp protein revealed that the protein is stable and can form hydrogen bonds with other proteins. The RdRp protein is immunogenic as well as non-allergenic and non-toxic. The results of our studies also corroborate the previous studies based on bioinformatics approach for the development of novel drugs and vaccine candidate to curb this deadly pandemic disease [38–41].

Despite the high efficacy and various advantages of peptide vaccines, they have not been licensed yet for human use; this is because peptides are very poor immunogens and hence require adjuvants (immune stimulator) to enhance immunogenicity or at least a suitable delivery system. Additionally, they are highly susceptible to enzymatic degradation because they represent a very short stretch of amino acids. The limitations and side effects of peptide vaccines include the lack of prolonged immune response, immune evasion, and localized immune stimulus [14, 42]. The use of carrier molecules might be inducing allergenicity and/or reactogenicity that enhances the complexity of peptide-based vaccine design.

In the present investigation, using various tools of immunoinformatics, multiple epitope-based vaccine candidates were predicted which are capable of stimulating both humoral as well as cellular immune response, as predicted vaccine construct possess both B-cell and T-cell epitopes accompanied with adjuvants. Thus, our *in silico* designed vaccine is suggested as a potentially good candidate for SARS-CoV-2 infections. Further, *in vitro* and *in vivo* studies are necessary to validate the designed vaccine construct.

## 5. Conclusion

The emergence of novel coronavirus pandemic poses an unprecedented threat to mankind globally; therefore, we urgently need efficient COVID-19 vaccines and therapeutics. RdRp of SARS-CoV-2 is able to stimulate a considerable amount of immunogenicity, since it exhibits lower glycosylation density as compared to structural proteins; hence, it may be used as an effective and durable vaccine candidate. Altogether, the results of the present *in silico* study revealed that the identified multi-epitope vaccine candidate of RdRp has immense potential to induce both innate and adaptive immune response against SARS-CoV-2. This epitope-based peptide vaccine exhibits drug-like properties compared to whole organism or recombinant protein vaccines, which is easy in vaccine delivery in the host and also opens up new avenues for molecular medicines to become a reality in the future. Moreover, our study showed high efficacy of designed multi-epitopes vaccine candidates using predictive tools of immunoinformatics; further, *in vivo* studies in the model organisms are necessary to better understand immunogenicity to validate the designed candidate vaccine.

## Figures and Tables

**Table 1:** Physicochemical properties of RdRp protein (wild type).

Physicochemical properties	RdRp	Amino acid composition	No.	Percent composition (%)
Molecular weight	106660.24	Ala (A)	64	6.9
No. of amino acids	932	Arg (R)	43	4.6
Theoretical pI	6.14	Asn (N)	56	6
Instability index	28.32	Asp (D)	75	8
No. of negatively charged (Asp + Glu)	106	Cys (C)	29	3.1
No. of positively charged (Arg + Lys)	94	Gln (Q)	28	3.0
Aliphatic index	78.43	Glu (E)	31	3.3
Grand average of hydropathicity	−0.224	Gly (G)	45	53
Estimated half-life (mammalian reticulocytes, in vitro)	1.9 hours	His (H)	27	2.9
Atomic composition		Ile (I)	33	3.5
C	4792	Leu (L)	83	8.9
H	7265	Lys (K)	51	5.5
N	1259	Met (M)	25	2.7
O	1401	Phe (F)	257	6.1
S	54	Pro (P)	30	3.2
Formula	C_4792_H_7265_N_1259_O_1401_S_54_	Ser (S)	53	5.7
Total number of atoms	14771	Thr (T)	61	6.5
		Trp (W)	9	1
		Tyr (Y)	58	6.2
		Val (V)	74	7.9
		Phy (O)	0	0.0
		Sec (U)	0	0.0

**Table 2:** B-cell epitope prediction of RdRp protein sequence of SARS-CoV-2 Indian isolate and its allergenicity.

No.	Start	End	Peptide	Length	Allergenicity
1	8	28	LNRVCGVSAARLTPCGTGTST	21	Allergen
2	39	44	NDKVAG	6	Non-allergen
3	58	64	EKDEDDN	7	Non-allergen
4	76	88	TFSNYQHEETIYN	13	Non-allergen
5	91	91	K	1	Non-allergen
6	95	96	AV	2	Non-allergen
7	106	113	IDGDMVPH	8	Non-allergen
8	136	139	EGNC	4	Non-allergen
9	154	169	DDYFNKKDWYDFVENP	16	Allergen
10	211	231	DLNGNWYDFGDFIQTTPGSGV	21	Non-allergen
11	257	283	VDTDLTKPYIKWDLLKYDFTEERLKLF	27	Allergen
12	288	303	KYWDQTYHPNCVNCLD	16	Allergen
13	318	326	STVFPPTSF	9	Non-allergen
14	360	366	NLHSSRL	7	Non-allergen
15	386	390	NLLLD	5	Non-allergen
16	405	436	VAFQTVKPGNFNKDFYDFAVSKGFFKEGSSVE	32	Non-allergen
17	444	463	QDGNAAISDYDYYRYNLPTM	20	Non-allergen
18	482	525	CYDGGCINANQVIVNNLDKSAGFPFNKWGKARLYYDSMSYEDQD	44	Non-allergen
19	533	533	R	1	Non-allergen
20	536	537	IP	2	Non-allergen
21	547	552	AISAKN	6	Non-allergen
22	596	597	GG	2	Non-allergen
23	599	621	HNMLKTVYSDVENPHLMGWDYPK	23	Non-allergen
24	644	648	TCCSL	5	Allergen
25	676	685	KPGGTSSGDA	10	Non-allergen
26	712	718	GNKIADK	7	Non-allergen
27	731	742	LYRNRDVDTDFV	12	Allergen
28	771	772	AS	2	Non-allergen
29	798	812	KCWTETDLTKGPHEF	15	Allergen
30	832	832	P	1	Non-allergen
31	834	834	P	1	Non-allergen
32	847	850	IVKT	4	Non-allergen
33	871	877	KHPNQEY	7	Allergen
34	893	893	D	1	Non- allergen
35	910	919	DNTSRYWEPE	10	Allergen
36	922	928	EAMYTPH	7	Allergen

**Table 3:** T-cell epitope prediction of SARS- CoV-2 RdRp protein sequence from India and its allergenicity.

Peptide	Length	Score	Allergenicity
PDILRVYANLGERVRQALLKTVQFCDAMRNAGIVGVLTLDNQDLNGNWYDFGDFIQTTPG	60	0.92466	Non-allergen
YWDQTYHPNCVNCLDDRCILHCANFNVLFSTVFPPTSFGPLVRKIFVDGVPFVVSTGYHF	60	0.65772	Allergen
LPYPDPSRILGAGCFVDDIVKTDGTLMIERFVSLAIDAYPLTKHPNQEYADVFHLYLQYI	60	0.56968	Non-allergen
SADAQSFLNRVCGVSAARLTPCGTGTSTDVVYRAFDIYNDKVAGFAKF	48	0.31069	Non-allergen
DMVPHISRQRLTKYTMADLVYALRHFDEGNCDTLKEILVTYNCCDDDYFNKKDWYDFVEN	60	0.29818	Allergen
LKTNCCRFQEKDEDDNLIDSYFVVKRHTFSNYQHEETIYNLLKDCPAVAKHDFFKFRIDG	60	0.28239	Allergen
RKLHDELTGHMLDMYSVMLTNDNTSRYWEPEFYEAMYTPHTVLQ	44	0.16705	Non-allergen
SGVPVVDSYYSLLMPILTLTRALTAESHVDTDLTKPYIKWDLLKYDFTEERLKLFDRYFK	60	0.15884	Non-allergen
STDGNKIADKYVRNLQHRLYECLYRNRDVDTDFVNEFYAYLRKHFSMMILSDDAVVCFNS	60	0.12277	Allergen
TVKPGNFNKDFYDFAVSKGFFKEGSSVELKHFFFAQDGNAAISDYDYYRYNLPTMCDIRQ	60	0.01508	Non-allergen
LLFVVEVVDKYFDCYDGGCINANQVIVNNLDKSAGFPFNKWGKARLYYDSMSYEDQDALF	60	−0.14064	Non-allergen
RELGVVHNQDVNLHSSRLSFKELLVYAADPAMHAASGNLLLDKRTTCFSVAALTNNVAFQ	60	−0.32362	Non-allergen
SHRFYRLANECAQVLSEMVMCGGSLYVKPGGTSSGDATTAYANSVFNICQAVTANVNALL	60	−0.33005	Allergen
AYTKRNVIPTITQMNLKYAISAKNRARTVAGVSICSTMTNRQFHQKLLKSIAATRGATVV	60	−0.71249	Non-allergen
IGTSKFYGGWHNMLKTVYSDVENPHLMGWDYPKCDRAMPNMLRIMASLVLARKHTTCCSL	60	−1.00228	Allergen
TYASQGLVASIKNFKSVLYYQNNVFMSEAKCWTETDLTKGPHEFCSQHTMLVKQGDDYVY	60	−1.21139	Allergen

**Table 4:** Class I immunogenicity of RdRp protein of SARS-CoV-2.

Peptide	Length	Score
PDILRVYANLGERVRQALLKTVQFCDAMRNAGIVGVLTLDNQDLNGNWYDFGDFIQTTPG	60	0.92466
YWDQTYHPNCVNCLDDRCILHCANFNVLFSTVFPPTSFGPLVRKIFVDGVPFVVSTGYHF	60	0.65772
LPYPDPSRILGAGCFVDDIVKTDGTLMIERFVSLAIDAYPLTKHPNQEYADVFHLYLQYI	60	0.56968
SADAQSFLNRVCGVSAARLTPCGTGTSTDVVYRAFDIYNDKVAGFAKF	48	0.31069
DMVPHISRQRLTKYTMADLVYALRHFDEGNCDTLKEILVTYNCCDDDYFNKKDWYDFVEN	60	0.29818
LKTNCCRFQEKDEDDNLIDSYFVVKRHTFSNYQHEETIYNLLKDCPAVAKHDFFKFRIDG	60	0.28239
RKLHDELTGHMLDMYSVMLTNDNTSRYWEPEFYEAMYTPHTVLQ	44	0.16705
SGVPVVDSYYSLLMPILTLTRALTAESHVDTDLTKPYIKWDLLKYDFTEERLKLFDRYFK	60	0.15884
STDGNKIADKYVRNLQHRLYECLYRNRDVDTDFVNEFYAYLRKHFSMMILSDDAVVCFNS	60	0.12277
TVKPGNFNKDFYDFAVSKGFFKEGSSVELKHFFFAQDGNAAISDYDYYRYNLPTMCDIRQ	60	0.01508
LLFVVEVVDKYFDCYDGGCINANQVIVNNLDKSAGFPFNKWGKARLYYDSMSYEDQDALF	60	−0.14064
RELGVVHNQDVNLHSSRLSFKELLVYAADPAMHAASGNLLLDKRTTCFSVAALTNNVAFQ	60	−0.32362
SHRFYRLANECAQVLSEMVMCGGSLYVKPGGTSSGDATTAYANSVFNICQAVTANVNALL	60	−0.33005
AYTKRNVIPTITQMNLKYAISAKNRARTVAGVSICSTMTNRQFHQKLLKSIAATRGATVV	60	−0.71249
IGTSKFYGGWHNMLKTVYSDVENPHLMGWDYPKCDRAMPNMLRIMASLVLARKHTTCCSL	60	−1.00228
TYASQGLVASIKNFKSVLYYQNNVFMSEAKCWTETDLTKGPHEFCSQHTMLVKQGDDYVY	60	−1.21139

## Data Availability

The data used to support the findings of this study are available from the corresponding author upon request.
